# EGFR trafficking: effect of dimerization, dynamics, and mutation

**DOI:** 10.3389/fonc.2023.1258371

**Published:** 2023-09-11

**Authors:** Destiny F. Schultz, Daniel D. Billadeau, Seetharama D. Jois

**Affiliations:** ^1^ Department of Immunology, Mayo Clinic, Rochester, MN, United States; ^2^ Division of Oncology Research, Mayo Clinic, Rochester, MN, United States; ^3^ Department of Pathobiological Sciences, School of Veterinary Medicine, Louisiana State University, Baton Rouge, LA, United States

**Keywords:** EGFR trafficking, dimerization, cancer, clathrin-mediated endocytosis, lysosome

## Abstract

Spontaneous dimerization of EGF receptors (EGFR) and dysregulation of EGFR signaling has been associated with the development of different cancers. Under normal physiological conditions and to maintain homeostatic cell growth, once EGFR signaling occurs, it needs to be attenuated. Activated EGFRs are rapidly internalized, sorted through early endosomes, and ultimately degraded in lysosomes by a process generally known as receptor down-regulation. Through alterations to EGFR trafficking, tumors develop resistance to current treatment strategies, thus highlighting the necessity for combination treatment strategies that target EGFR trafficking. This review covers EGFR structure, trafficking, and altered surface expression of EGFR receptors in cancer, with a focus on how therapy targeting EGFR trafficking may aid tyrosine kinase inhibitor treatment of cancer.

## Introduction

Epidermal growth factor receptor (EGFR) is largely considered to be the most well-studied receptor tyrosine kinase (RTK) (1–3). Following the initial discovery of epidermal growth factor (EGF) by Stanley Cohen in 1963, the EGFR family was identified as receptors for EGF (4, 5). Consisting of four members, ErbB1-4 (human epidermal growth factor receptors HER1-4), both homo- and hetero-dimerization of receptors within this family lead to downstream signaling. So far, eight EGFR (HER1) ligands have been reported. However, there are no known ligands for EGFR family member HER2(6–10) despite reports that HER2 undergoes dimerization with other EGF receptors and generates signals for cell growth (11). Importantly, HER2 overexpression and mutation have been observed in many human cancers and the presence of these abnormalities can determine clinical treatment [reviewed in (12)].

**Figure 1:**
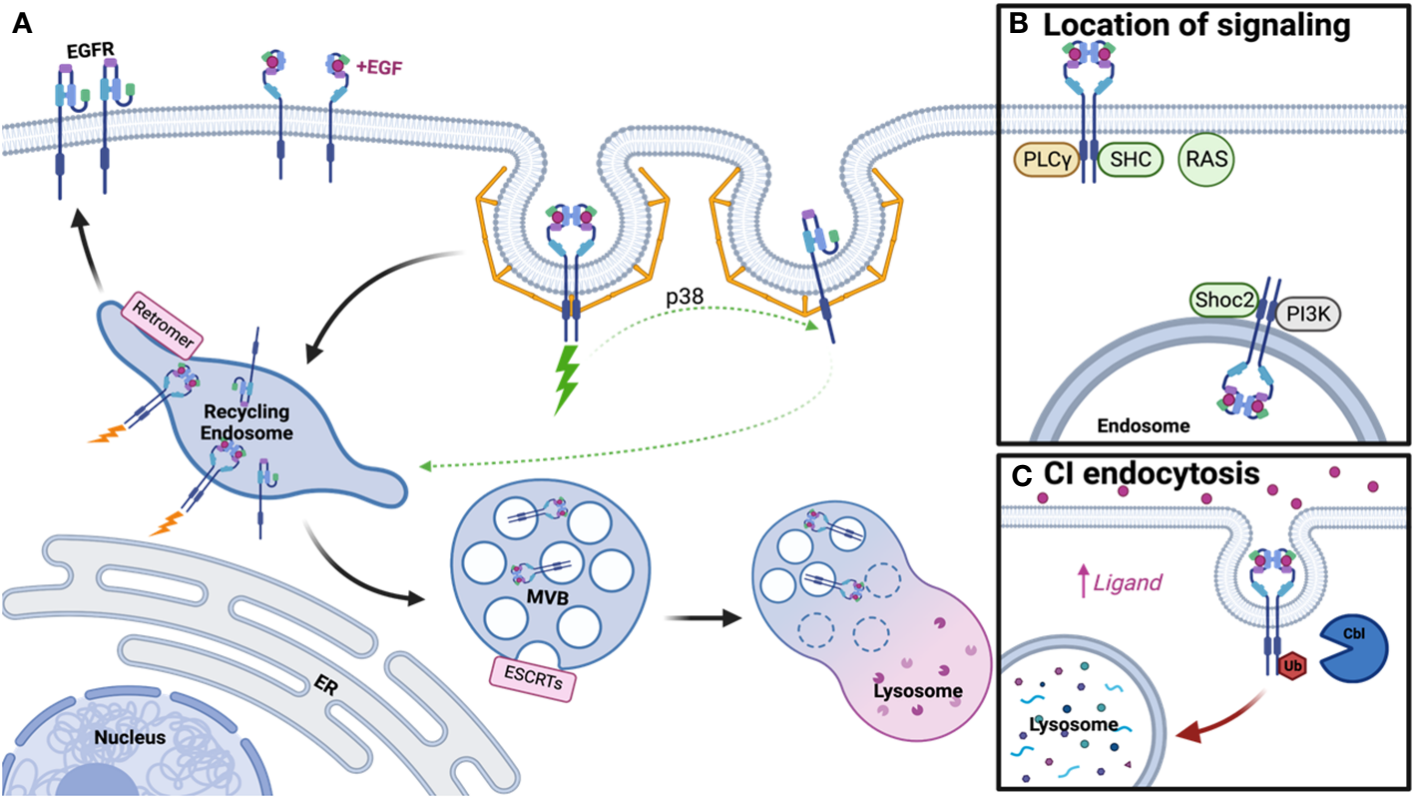
Schematic of EGFR Trafficking and Signaling. **(A)** Clathrin-mediated endocytosis of EGFR dimers and monomers under low ligand conditions promotes receptor recycling, while ubiquitinated dimers will be degraded. **(B)** Distinct signaling adaptors are associated with EGFR dimers at the plasma membrane and endosome. **(C)** Clathrin-independent endocytosis occurs under high ligand concentration, promotes receptor ubiquitination, and subsequent lysosomal degradation. Created with BioRender.com.

Signaling pathways engaged by the EGFR family regulate cell growth, differentiation, invasion, and wound healing. The signal transduction mechanism is tightly regulated by ligand binding to extracellular domains (ECD) of EGFRs, resulting in a change in conformation and dimerization, passing the signal from outside the cell to the cytoplasmic side via the transmembrane domain and finally cross-phosphorylation by the cytosolic kinase domain (1–3). Adapter proteins help carry out the downstream signaling events leading to the activation of transcription factors for cell growth. Signaling is terminated by receptor downregulation, whereby active receptors undergo endocytosis and are sorted into the lysosome for degradation following ubiquitination. However, some of the receptor molecules are recycled back to the cell surface to maintain the number of EGF receptors on the surface in a process termed EGFR recycling (13–16) (**Figure 1**). The disruption of proper EGFR signaling and trafficking leads to diseases like cancer, where overexpression or activating mutations within EGFR promote tumor growth. Current treatments for these types of cancers include tyrosine kinase inhibitors (TKIs), which inhibit downstream signaling by directly impairing EGFR tyrosine kinase activity. However, many patients have tumors that become non-responsive to TKIs, thus drawing the need for new treatment strategies in EGFR-driven tumors (17–19).

In the present review, we summarize the role that EGFR structure, dimerization, and trafficking play in signal modulation (**Figure 1**). Further, we discuss how targeting of these processes in combination may be able to overcome current treatment limitations for tumors that overexpress EGFR or have an EGFR-activating mutation.

## Biochemical basis of activation

**Figure 2:**
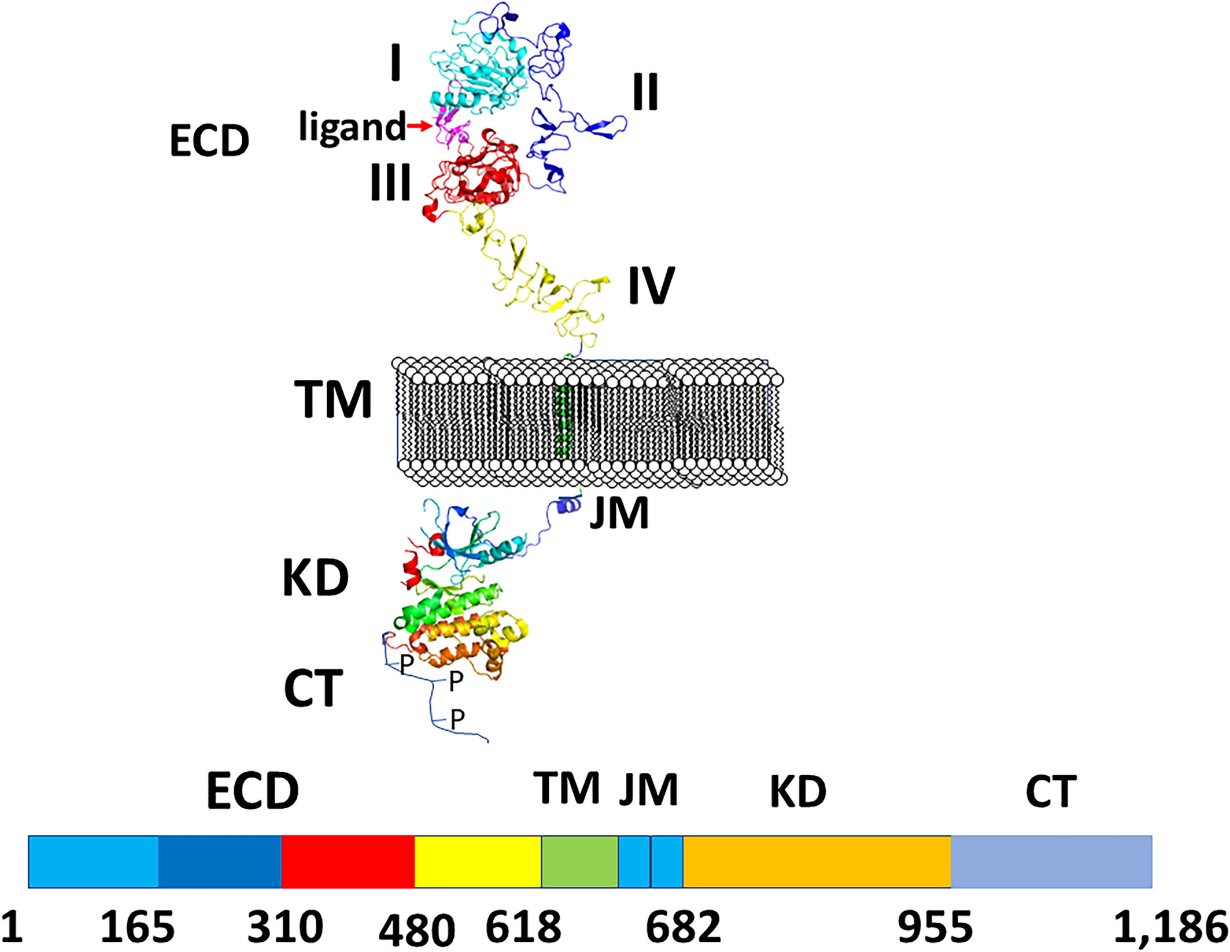
A schematic diagram of EGFR structures showing monomer with different domains labeled. ECD, extracellular domain, TM, a transmembrane domain, KD, kinase domain, JM, juxtamembrane domain, CT, cytoplasmic domain. Carboxy terminal tail is shown as unstructured with P indicating phosphorylation site. Crystal structures of different domains were used to generate the structure of EGFRs. PDB ID: 3NJP (20), 2KS1 (21), 3GOP (22).

**Figure 3:**
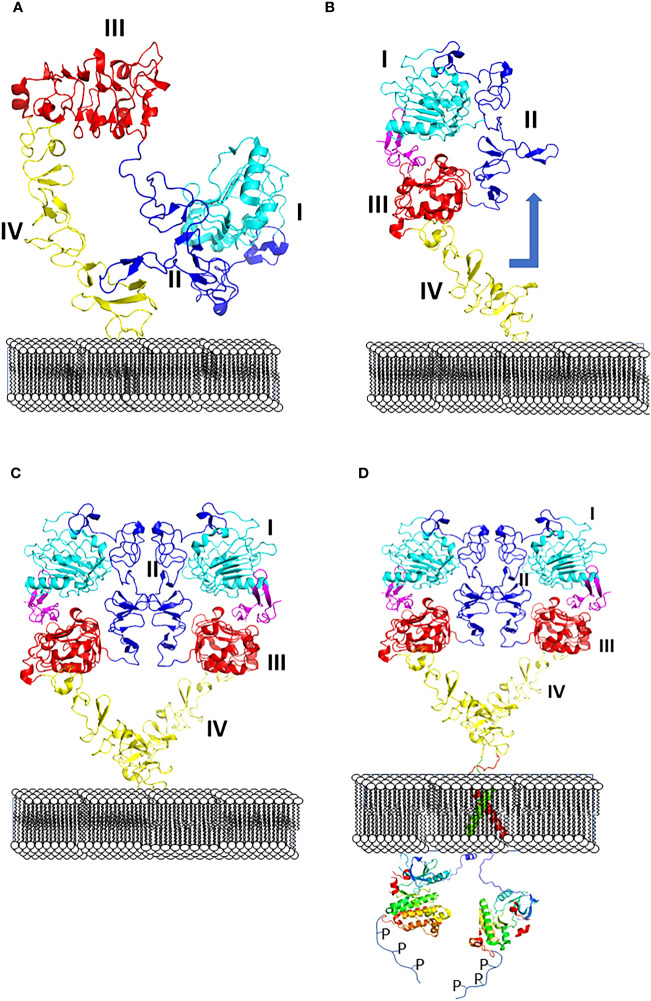
**(A)** Closed (left) and **(B)** open (right) conformation of EGFR extracellular domain and changes in conformation with respect to the membrane surface. **(C)** Upon change in conformation monomers of ECD of EGFR form dimers, dimer interface with domains I to IV are shown. **(D)** Dimerization of ECD results in phosphorylation of kinase domain by changes in transmembrane domain and kinase domain activation. PDB ID:1NQL (2) and 3NJP (20), 2KS1 (21), 3GOP (22).

All four EGF receptors have a similar structure, with a nearly 620 amino acid extracellular domain (ECD), short transmembrane domain (TM), juxtamembrane domain (JM), a 540 amino acid intracellular region containing the kinase domain (KD), and a carboxy-terminal tail made of 230 amino acids with multiple phosphorylation sites (**Figure 2**). The ECD consists of domains I to IV: I and III are involved in ligand binding, and domains II and IV are cysteine-rich domains and contain a string of disulfide bonds. Structures of EGFR “open” and “closed” conformations have been elucidated by X-ray crystallography (9, 20, 21). In the closed (tethered) conformation (**Figure 3**), domain II forms contact with domain IV, thereby blocking any other molecular contact with domain II. Domains I and III form a huge groove on one side of the molecule that can be occupied by EGFR ligands. Upon ligand binding, domains I and III come close, promoting the extended conformation in which domain IV moves away from domain II, thus opening domain II and IV for interaction of its dimerization partner (open conformation). Different possible dimers of EGFR (e.g., EGFR-EGFR, EGFR-HER2, HER2-HER3) have a similar extracellular dimer structure (**Figure 3**). Importantly, HER2, which is not known to interact with any EGF ligands, exists in an open conformation, thus allowing it to partner with other EGFR molecules that have bound ligand. Dimerization of the ECD then induces dimerization of the TM helical region through N-terminal GxxxG-like motifs. Further, the JM domain also contacts its partner EGFR molecule resulting in asymmetric dimerization of the kinase domain. EGFRs that are studied in detergent micelles suggest that the dimerization of ECD does not necessarily lead to the dimerization of the kinase domains (23, 24). The kinase domain contains C and N-lobes; upon dimerization, the C-lobe of one kinase interacts with the N-lobe of the dimerization partner’s kinase allowing for cross-phosphorylation. This results in downstream signaling through the recruitment of adaptor proteins to the phosphorylated tyrosine residues (25). Although the biochemical action of EGFR is described at the molecular level, most of the information about EGFR structure and mechanism of action is based on the structure of individual domains since full-length EGFR structure is difficult to elucidate with available experimental methods. Therefore, one has to put the available structural pieces together to get the purported overall structure of full-length EGFR family members, and to infer mechanisms contributing to their dimerization and signaling in cells.

The ECD of EGFRs is known to dimerize upon ligand addition and induce the dimerization of the intracellular kinase domain, however, there are reports that isolated kinase domains dimerize and activate signaling when JM segments are present (22, 26, 27). Therefore, it is proposed that ligand-free EGFRs can undergo dimerization and exist as active and inactive dimers. In support of this notion is the observation that overexpression of wild type EGFR leads to ligand-independent activation of IRF3, rather than the ERK or AKT pathways, which has been termed non-canonical EGFR signaling (28). This has been further supported by changes in distribution within the plasma membrane, with high EGFR expression promoting oligomerization and ligand-independent phosphorylation that appears to have no impact on ERK or AKT signaling (28, 29). In addition to EGF-induced and ligand-independent activation, EGFR activation can be induced by six other ligands (30). These EGFR ligands have a varying affinity, induce specific dimerization pairings of the four EGFRs, and lead to distinct cellular outcomes. Thus, activation of EGFR signaling can occur in scenarios outside of EGF addition, which leads to specific receptor dimerization/oligomerization and varying cellular response.

The plasma membrane also plays a role in modulating EGFR dimerization, activation, and autoinhibition. Within the plasma membrane, cholesterol-rich lipid microdomains have been found to promote quick movement of EGFR and HER2, which allows for rapid dimerization and signaling upon ligand addition (31–34). Conversely, depletion of membrane cholesterol through methyl-β-cyclodextrin treatment leads to the accumulation of EGFR within confined regions of the membrane and therefore promotes ligand-independent receptor activation (31). While these findings were in live cells using microscopic techniques, they have limitations in terms of what EGFR is doing at a structural level within the membrane that recent molecular dynamics (MD) simulations have started to fill in.

The EGFR kinase domain surface has many basic residues (27), which are shielded by the C-terminal domain in the active dimer. MD simulations suggest that the EGFR kinase domain is attached to the membrane by interaction of the basic residues with anionic lipids (35). Thus, the active site of the kinase domain is not exposed. On the other hand, asymmetric dimers of kinases have less interaction with anionic lipids, and the kinase domain is available for phosphorylation, making EGFR active (36). Although EGFR ECDs are assumed to be perpendicular to the plane formed by the cell membrane, MD simulations studies found that the ECD of EGFR molecules lie down on the membrane. This orientation brings the EGF-binding site adjacent to the membrane surface where it can interact with the membrane-bound ligand (37). This asymmetric nature of EGFR dimers, where one of the ligands is bound to the membrane as well as EGFR and the other only bound to EGFR, may lead to negative cooperativity. Although MD simulations provide some insight into the dynamic nature of EGFR receptors and their dimers, these structures are modeled based on X-ray crystal structure and solution structure using NMR and modeling methods. In reality, dimerization and activation of the kinase domain seem to depend on the microenvironment and charges of lipid head groups facing the cytoplasmic side of the membrane (24, 27, 35–37). Thus, signal transduction processes at the molecular level have yet to be elucidated in detail.

Moving forward, structural elucidation of full-length EGFRs using cryo-EM and molecular modeling may provide new insights into the signal transduction process of EGFR family members. Furthermore, EGFRs that lack part or full ECD have been found in clinical studies (38–40), calling into question the importance of ECD and conformational changes associated with ECD and TM for kinase activity. Thus, altered or mutated forms of EGFR exist in disease states of patients, which don’t fit into the current models, and hence extensive structural and functional studies of EGF family receptors are needed to address those limitations.

## Regulation of EGFR trafficking and signaling

In 1976, Carpenter and Cohen published the first paper on EGFR trafficking, postulating that EGFR-bound growth factor could enter human fibroblasts and be degraded within the lysosome (41). However, little was known about the regulation of this pathway and whether it served a purpose for receptor signaling. It is now appreciated that along the pathway to the lysosome, several steps participate in the regulation of EGFR trafficking and signaling, including endocytosis, protein recycling, and protein degradation (**Figure 1A**).

At the plasma membrane, active EGFR dimers generate a conformation that leads to the trans-phosphorylation of tyrosine residues in the cytoplasmic tail, promoting the recruitment of numerous signaling adaptor proteins that engage pathways such as the RAS-MAPK cascade and the phosphoinositide-3-kinase (PI3K) pathway. In addition to signaling adaptors, phosphorylation of EGFR at Tyrosine 1068 and 1086 leads to growth factor receptor–bound protein 2 (Grb2) binding, thus promoting the recruitment of adaptor protein complex-2 (AP-2) and allowing EGFR to undergo clathrin-mediated endocytosis (CME) (42, 43). CME is also regulated by ubiquitination and acetylation of EGFR (44), with clathrin acting as a signaling scaffold for the AKT pathway (45). However, recent literature suggests endocytosis of EGFR is more complicated than the canonical CME model, with subsets of clathrin-coated pits that may not require AP-2, and instead rely on other endocytic adaptors, to promote endocytosis (46, 47). A further layer of complexity to EGFR endocytosis is added when ligand concentration is taken into consideration. In contrast to low concentration of ligand, which promotes CME, high concentration of ligand promotes ubiquitination of active EGFR by the E3 ubiquitin ligase Cbl, thus allowing it to undergo rapid clathrin-independent endocytosis (CIE) and steering it toward eventual degradation (46). Thus, a simplified model of EGFR dimer activation and endocytosis has been generated, however this fails to address any further clustering of active dimers and endocytosis of EGFR monomers.

Unlike the simplified model, which only requires phosphorylation of a dimer for signaling to occur, a more complicated model emerges whereby this activation depends on ligand addition and further oligomerization of EGFR (48, 49). Under low ligand concentration, it’s been thought that ligand-bound dimerized EGFR can trans-phosphorylate nearby dimers that are unbound, thus amplifying the signal (48). However, this model is apart from the traditional endocytosis model and more work is needed to elucidate if endocytosis of all receptors within the oligomer would occur in a CME manner. In contrast to oligomers, more work has been done on how inactive monomers internalize. Unbound EGFRs are internalized at a slower rate compared to ligand-bound EGFRs (50), which may in part be due to endocytic regulation based on signals from active dimers. Active EGFR triggers p38 activation, which phosphorylates EGFR monomers near Serine 1015, resulting in CME (51). Thus, ligand concentration and receptor activation are closely linked to the regulation of endocytosis for both active dimers and unbound monomers.

Following endocytosis, EGFR traffics to the early endosome, where decisions are then made for receptor recycling or degradation. Apart from these pathways, EGFRs are also known to be transported into the nucleus after early endosomal sorting to participate in transcriptional regulation (52, 53). While signaling is initiated at the plasma membrane by ligand binding and dimerization, whether the receptor continues to signal from the endosome remains highly debated. Early literature suggested active EGFR continued to signal from the endosome until incorporation into intralumenal vesicles (ILVs), thus inhibiting access of the EGFR C-terminus to cytosolic signaling effectors. The first study to assess the relationship between signaling and cellular location utilized mutant dynamin and proposed that phospholipase C gamma (PLCγ) and Shc signaling occur at the plasma membrane while further EGFR phosphorylation, ERK, and PI3K signaling derive from the endosome (54). Further literature suggested this may be in part due to which adaptors are associated with EGFR at these cellular locations, with some only interacting at the plasma membrane or intracellularly and others appearing in both populations (55). Some adaptors appeared to traffic from the plasma membrane to the endosome with EGFR upon EGF addition and sustain signaling throughout this process (56–58) and adaptors/scaffolds that aid in signaling at the endosome, such as Shoc2, have also been reported (59). Utilizing spatial proteomics, many signaling molecules have also been found in proximity to endosomal EGFR (60). However, the depletion of dynamin in mouse fibroblasts suggested signaling was unimpacted and therefore a majority of EGFR signaling may occur at the plasma membrane (61). Further, RAS primarily localizes to the plasma membrane and not the endosome, thus limiting where EGFR signaling mediated by RAS can occur (62, 63). These studies highlight that while some adaptors traffic with EGFR to the endosome, signaling may be limited by localization of other required components to the plasma membrane (**Figure 1B**). Thus, the debate on where signaling occurs is still ongoing but may depend on factors like cell type, protein depletion, method of overexpression, or technical limitations.

While at the endosome, one potential fate for EGFR is recycling back to the plasma membrane, which is mediated by the Retromer, Retriever, COMMD/CCDC22/CCDC93 (CCC), and WASP and SCAR homologue (WASH) complexes (64). Depletion of these complexes promotes lysosomal degradation of cargo (65, 66) thus suggesting more of an active process for cargo selection in recycling than previously appreciated (59). For unbound EGFR monomers, this recycling is regulated by p38-mediated phosphorylation either downstream of low-concentration EGF addition (51) or phosphatidic acid signaling (67), though it remains unclear which recycling machinery is necessary for this process. On the way back to the plasma membrane, it’s also thought that any spontaneously active monomers are shut off by protein tyrosine phosphatase 1B (PTP1B)-mediated dephosphorylation (68). In addition to active cargo selection, proteins that inhibit selection for degradation, like lipocalin-2, also induce recycling and sustained EGFR activity (69). Outside of recycling logistics for monomers, most research on EGFR recycling and signaling has been conducted in the context of benefitting cancer growth and progression, as discussed later.

Alternatively, endosomal EGFR may undergo selection for lysosomal degradation. Entrance into the degradation pathway is reliant upon EGFR ubiquitination, by ligases such as Cbl and ZNRF1, thus allowing recognition by the endosomal sorting complexes required for transport (ESCRTs) for incorporation into ILV (59, 70–73). Subsequent fusion of the multivesicular body (MVB) with the lysosome leads to EGFR degradation (74). Mutations in the ESCRT pathway have been associated with endosomal EGFR accumulation and enhanced signaling, but delayed receptor turnover, thus providing some support for the endosomal signaling model. Post-translational modification of the ESCRT machinery, such as glycosylation of HRS/HGS, has also been shown to play a role in regulation of EGFR degradation and signaling (75). It’s been suggested that ligand concentration/method of internalization dictates receptor fate, with clathrin independent endocytosis under high ligand concentration leading to receptor degradation (**Figure 1C**). Interactions with the ER may provide some cues for determining EGFR fate, with ER resident proteins able to keep the endosome in the perinuclear region of the cell to promote degradation and signal termination (76, 77). Thus, degradation and signaling are not only mediated by the ESCRT machinery, but also by intracellular localization of the endosomal compartment.

Spatially, the recycling and degradation machinery reside in different microdomains within the endosome, and a present area of focus is how cargo moves into the proper microdomain. Thus far, there have been studies linking recycling machinery such as WASH and receptor-mediated endocytosis -8 (RME-8) to recruitment and activity of ESCRT-0 component HRS, though no direct interaction has been found (78, 79). Based on actin nucleation being regulated by WASH, the transition to ESCRT-0 and choice between cargo recycling and degradation may be partially dependent upon actin binding/recognition. Further, RME-8 interacts with Hsc70 to disassemble endosomal clathrin, which is thought to be critical for assembly of HRS and transition to a degradative microdomain (80, 81). Thus, coats of either actin or clathrin play a role in determining the endosomal microdomain which cargo is incorporated into and therefore fate of the cargo. In addition to these coats providing a microdomain platform, studies in plants have shown a direct interaction between ESCRT-associated ALIX and retromer subunits (82), thus suggesting there may be additional processes/mechanisms by which these microdomains are regulated. Cumulatively, these microdomains are a continued area of study which may provide the ability to modulate trafficking and signaling of receptor cargo.

## Limitations of EGFR-mediated cancer treatment strategies

Tyrosine kinase inhibitors (TKIs) serve as the main therapy to target EGFR in cancer and have evolved through many generations. Imatinib was the first TKI approved for cancer therapy just over two decades ago (83) and was followed by first-generation EGFR-specific TKIs, gefitinib and erlotinib, thus revolutionizing targeted therapy by kinase inhibitors (84). Presently, there are four generations of TKIs, with those up through the third being approved for clinical use (**Table 1**) (104, 105). However, TKIs only delay tumor growth, and most tumors develop resistance within two years due to intrinsic or acquired changes.

**Table 1:** Representative EGFR tyrosine kinase inhibitors from each generation to overcome resistance and mutations of EGFR kinase domain.

TKI/year of approval	Structure	Targeted mutation*/*reversible, irreversible	Possible Resistance development	Reference
First generation				
Erlotinib 2004	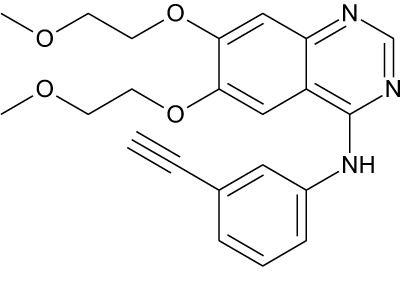	Glu746-Ala750 deletion in exon 19 and the common p.Leu858Arg substitution in exon 21 Reversible	EGFR T790M HER2 amplification	(84)
Gefitinib 2003	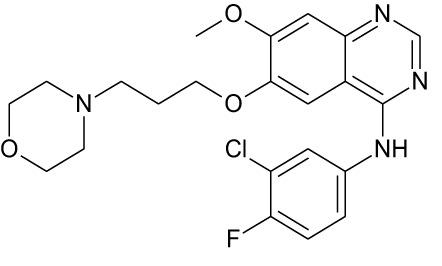	Glu746-Ala750 deletion in exon 19 and the common p.Leu858Arg substitution in exon 21 Reversible	EGFR T790M HER2 amplification	(85, 86)
Lapatinib 2007	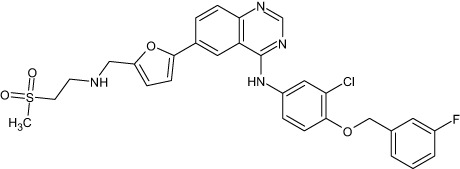	Targets both EGFR and HER2 kinase Reversible	Overexpression with activation of other tyrosine receptor kinases Axl, MET, IGF-1R, VEGF	(87)
Second generation				
Afatinib 2013	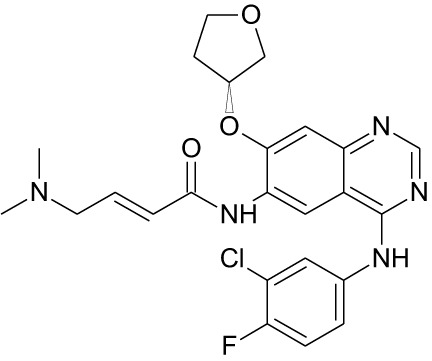	Del19/L858R uncommon *EGFR* mutations (S768I/G719X/L861Q) Irreversible	EGFR T790M HER2 amplification MET amplification	(88)
Dacomitinib 2018	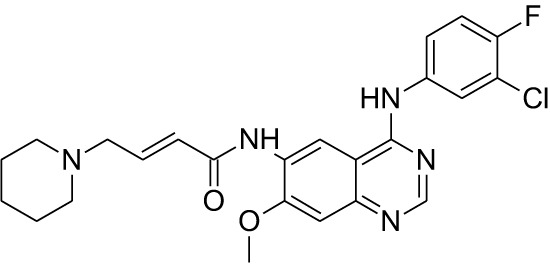	EGFR exon 19 deletion or exon 21 L858R substitution mutations Irreversible	EGFR T790M HER2 amplification MET amplification	(89) (90)
Neratinib 2017	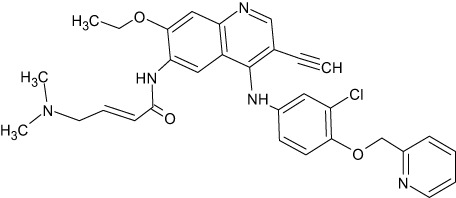	Targets EGFR, HER1, HER2, and HER4. Irreversible	*HER2* alteration, alterations in the HER3/PI3K/protein kinase B, AKT (mTOR) and MAPK signaling.	(91–93)
Third generation				
Rociletinib 2022	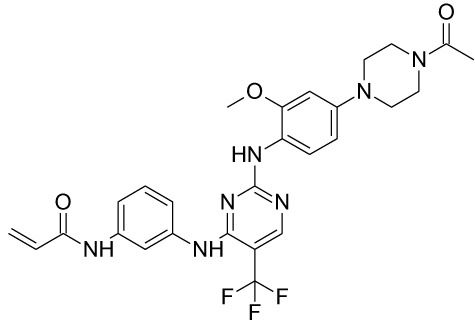	EGFR Ex19del L858R, T790M Irreversible	EGFR C797S HER2 amplification, MET amplification KRAS mutation	(94) (95)
Osimertinib 2015	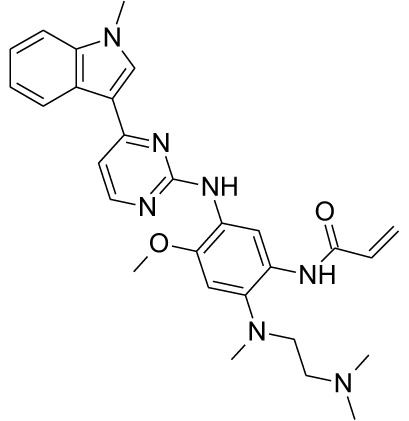	*EGFR T790M and EGFR sensitive L858R and Del19* Irreversible	EGFR C797S MET amplification HER2 amplification/mutation PIK#CA Amplification/mutation	(96) (97)
Olmutinib 2016	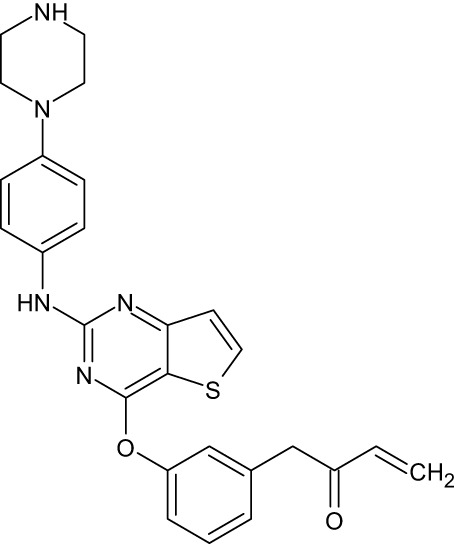	EGFR Ex19del L858R, T790M Irreversible	EGFR C797S	(98, 99)
Fourth generation or Multi-target TKIs				
Brigatinib 2017	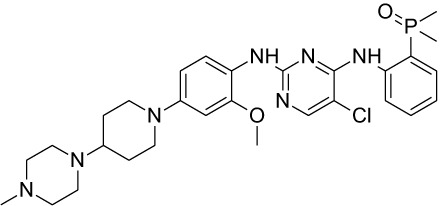	EGFR Ex19de L858R, C797S T790M Reversible	Possible *NTRK* rearrangement (*LIPI-NTRK1*)	(100) (99)
Vandetanib 2011	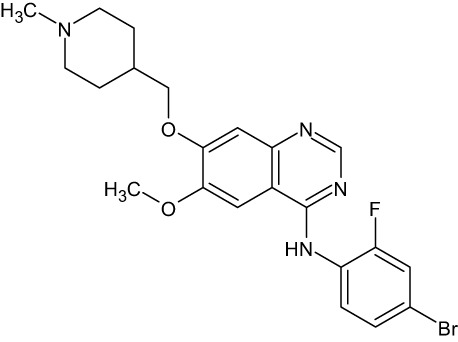	VEGFR, EGFR and RET tyrosine kinases. Reversible	Substitution at codon 904 in the activation loop of the RET kinase domain	(101–103)

Structure of TKIs were drawn based on structures in Selleckchem.com.

Intrinsic resistance can be caused by genetic aberrations in multiple cancer signaling pathways, as is the case non-small cell lung cancer (NSCLC) patients with EGFR T790 mutation who do not respond to gefitinib and instead maintain proliferative and cell survival signaling in the presence of TKIs using alternative pathways such as integrin signaling (106, 107). It was shown that interaction of EGFR and integrin β4 can affect the sensitivity of gefitinib treatment in gastric cancer (108). Third generation TKIs, such as Osimertinib, have been developed to treat these patients (109), however they develop drug resistance through unknown mechanisms (110). Fourth generation inhibitors or multi-targeting TKIs such as brigatinib and vandetanib are developed to overcome the resistance developed by third generation TKIs. However, brigatinib therapy seems to develop resistance due to NTRK rearrangement in some patients (99–103). A small-molecule EGFR inhibitor, ERAS-801 has received an orphan drug designation by the FDA for malignant glioma. It is an orally available molecule that has significant CNS penetration (111). Another small molecule tucatinib, a reversible TKI, is known to bind to HER2 with specificity. Tucatinib in combination with antibody trastuzumab has shown efficacy in breast cancer and this drug was recently approved by FDA for the treatment of HER2 positive metastatic breast cancer (112, 113). Acquired resistance is caused by on-target secondary mutations and new generations of TKIs were developed to overcome the resistance (86, 114), but these still lack the ability to address drug resistance associated with altered receptor trafficking (115–117). For example, in a subset of NSCLC the mutant EGFRs preferentially recycle rather than degrade, leading to enhanced signaling by EGFR and the proto-oncogene tyrosine kinase Src (14, 116, 118–121). Cumulatively, further understanding and modulation of EGFR trafficking in cancer may provide an additional treatment option to bypass resistance to TKI treatment.

One of the mechanisms utilized by cancer cells to develop resistance is to avoid ubiquitin-mediated degradation of EGFR. In tumors harboring EGFR variant VIII, signaling is independent of ligand addition and remains constitutively active, however ubiquitination by the ubiquitin ligase Cbl promotes lysosomal degradation and therefore signal termination (122, 123). However, further EGFR mutations, which impair the interaction with Cbl, limit incorporation into MVBs for lysosomal degradation and instead cause prolonged signaling (124). Another way to avoid ubiquitin-mediated degradation is relocation of EGFR to the nucleus mediated by the tumor suppressor protein TIP30 (125, 126) and nuclear translocation signals within the juxtamembrane region (127), thus mutation at this level would also render TKI therapy ineffective and promote resistance.

Another mechanism of resistance is inhibition of EGFR downregulation by HER2. HER2 can act as an inhibitor of the downregulation of other EGFR family members due to the ECD existing in an open conformation thereby promoting receptor heterodimerization (2, 128–130). Overexpression of HER2 is thought to impact EGFR trafficking in two ways: decrease downregulation/internalization from the plasma membrane (131–135), and/or reroute internalized EGFR from the degradation pathway to the recycling pathway (136). Multiple proposed mechanisms exist for how HER2 may inhibit downregulation including inducing a conformation change, interactions with lipid raft components (137), and inhibition of clathrin coated pits (138, 139). In contrast, HER2 may promote recycling of EGFR since it isn’t contained in an endolysosomal compartment (138, 140–143) and is recycled back to the plasma membrane in a sortilin-related receptor 1 (SORLA)-dependent manner (144, 145). It’s also been thought that at least some regulation of HER2 trafficking is through the kinase domain, with binding of Hsp90 allowing for sequestration and preventing catalytic activity of HER2 (71, 146–148). Lastly, it was recently shown in breast cancer cells that pharmacologic inhibition of endosomal recycling using primaquine or knockdown of the Rab coupling protein led to the lysosomal accumulation and degradation of HER2 (149). These manipulations were found to synergize with anti-HER2 therapies and overcome resistance to the TKI lapatinib, thus suggesting that manipulating endosomal recycling could be a viable strategy to overcome resistance to therapies targeting the EGFR family of receptors.

Thus far, antibodies have been designed to target HER2 trafficking mechanisms, however these rely on HER2 being the main source of resistance for a tumor. One such antibody, Trastuzumab, has been found to inhibit ligand-independent activation, promote internalization, and prevent shedding of the HER2 ECD (150–153). In the case that a truncated form is present that lacks the ECD (p95HER2) (154–157), it’s thought that resistance to trastuzumab can develop, but thus far clinical studies have found no significant difference in survival among patients with p95HER2 relative to those lacking it (40, 158–160). Pertuzumab has also been shown to prevent dimerization of HER2 (161) and a combination with trastuzumab produces synergistic effects (162–166). Additionally, polyclonal, and bispecific anti-HER2 antibodies have been shown to promote rapid internalization and subsequent degradation of HER2 (165, 167, 168). These data show that HER2 can be targeted and regulate EGFR in cancer, however they fail to address how trafficking of EGFR, HER2, and HER3 impacts other receptors. EGFR trafficking in the absence of other family members has been extensively studied, but EGFR trafficking in these conditions is an ongoing area of research.

## Combining TKIs with EGFR trafficking inhibitors for cancer therapy

Since the main mechanism of resistance to TKI therapy involve vesicular trafficking, either by avoiding ubiquitin-mediated degradation or through HER2 trafficking, it stands to reason that a combination therapy approach could be effective. Preliminary understanding of efficacy in part derives from HER2 antibody studies, as discussed in the previous section, but also from existing TKIs that can alter trafficking of HER2 at high concentration. An example of this is Neratinib, a pan-HER TKI (169), which inhibits kinase activity and induces the internalization of EGFR/HER2 receptors through CME leading to their degradation only at a high dose (170). While adjusting TKI concentrations can be easily done in the context of tumor cell lines, it poses a potential toxicity problem when translating to humans. As such, further development of either TKIs that alter trafficking at low concentration or of small molecules that can be combined with TKIs is necessary.

One of the trafficking steps often dysregulated during TKI resistance in endocytosis, so preliminary studies on combination therapy have utilized molecules that inhibit CME. Through *in vitro* work and mouse models, it’s become appreciated that pairing the TKI gefitinib with endocytosis inhibitors decreases tumor cell survival (171, 172). Thus, a model has been formed that upon CME inhibition, EGFR undergoes macropinocytotic-dependent internalization, thereby promoting lysosomal degradation rather than receptor recycling (173). Depending on the molecule used to inhibit CME, mechanisms of internalization beyond macropinocytosis may also be utilized. This is the case with the small molecule DPBA, which mediates flotillin-dependent internalization through lipid rafts rather than relying on post-translational modification of EGFR (174), thus allowing it to work for WT or TKI resistant tumors. However, CME is just one step of EGFR trafficking and several other steps exist before EGFR degradation that may also be beneficial to target for combination therapy.

After endocytosis, EGFR moves through the endocytic system where it may continue to signal until incorporation into a MVB for degradation. Studies have suggested that ability to sustain EGFR signaling from endosomes promotes apoptosis (175), however the cell lines utilized are known to undergo EGFR-mediated death. Additional studies have supported the notion by knockdown of Neuropilin-2 leading to entrapment of EGFR in early endosomes and subsequent cell death (176), though the definitive linkage between EGFR signaling and cell death remains unclear. Targeting this step of trafficking may prove to be the most controversial approach to TKI resistance due to the unknowns on whether EGFR actually signals from endosomes. Further questions arise when considering that most endocytic machinery have been historically considered tumor suppressors, and the sustained EGFR signaling thought to be advantageous for survival. Thus, advanced understanding of endosomal EGFR signaling and how it may impact tumor viability are necessary before this strategy is viable for combination therapy with TKIs.

Finally, nuclear translocation of EGFR is another mechanism of TKI resistance that may be a useful target for combination therapy. Like the strategies above for keeping EGFR at endosomes to modulate signaling, strategies at this step of trafficking aim to inhibit nuclear translocation and therefore keep EGFR at endosomes. Early studies in cell lines have shown that small molecules, such as Primaquine and 1,25-dihydroxyvitamin D, block nuclear translocation and may promote changes in EGFR signaling and cell death (177, 178). However, limitations of these studies are apparent in that both treatments may have unintended consequences for other cell processes that make it hard to discern EGFR-dependence. Additionally, combination of these therapies with TKIs has not yet been assessed, therefore highlighting additional understanding required before these approaches can be translated to patients. Though many steps in EGFR trafficking exist that can be utilized to overcome resistance to TKI therapy, research on combination of TKIs with endocytosis inhibitors is the most advanced but still has a way to go before advancing to the clinic. Thus, combination of EGFR trafficking and TKIs presents an understudied area that has potential opportunity to benefit cancer patients with TKI resistance.

## Concluding remarks

The EGFR family of growth factor receptors remains the most extensively studied receptor family due to its clear association with cancer development and progression. Indeed, overexpression of these receptors, mutation, evasion of degradation, enhanced recycling, and/or altered signaling pathways of EGFR results in cancer development through enhancing downstream signaling. The generation of detailed structures of these receptors has provided important insight into the underlying molecular mechanisms contributing to receptor activation/dimerization and signal transduction. Moreover, they have provided atomic level detail on the mechanism of action of TKIs and monoclonal antibodies targeting the EGFR family of receptors. Many tyrosine kinase inhibitors have been developed as therapeutic agents for cancer. However, most EGFR-expressing tumors eventually become resistant to these inhibitors, thus requiring new treatment strategies. It is becoming clear that EGFR trafficking has implications in different types of cancer and that the development of resistance to TKIs is related to altered EGFR trafficking. Therefore, in addition to TKI therapy, EGFR/HER2 trafficking may be an additional target for cancer treatment. Findings in this area could increase efficacy and overcome resistance to TKI treatments that occur in the patient population.

## Author contributions

SJ: Conceptualization, Funding acquisition, Project administration, Resources, Writing – original draft, Writing – review & editing. DS: Writing – original draft, Writing – review & editing. DB: Conceptualization, Resources, Supervision, Writing – original draft, Writing – review & editing.

## References

[1] Burgess AW Cho HS Eigenbrot C Ferguson KM Garrett TP Leahy DJ. An open-and-shut case? Recent insights into the activation of EGF/ErbB receptors. Mol Cell (2003) 12:541–52. doi: 10.1016/S1097-2765(03)00350-2 14527402

[2] Ferguson KM. Structure-based view of epidermal growth factor receptor regulation. Annu Rev Biophys (2008) 37:353–73. doi: 10.1146/annurev.biophys.37.032807.125829 PMC2745238 18573086

[3] Lemmon MA Schlessinger J Ferguson KM. The EGFR family: not so prototypical receptor tyrosine kinases. Cold Spring Harb Perspect Biol (2014) 6:a020768. doi: 10.1101/cshperspect.a020768 24691965 PMC3970421

[4] Cohen S. Isolation of a mouse submaxillary gland protein accelerating incisor eruption and eyelid opening in the new-born animal. J Biol Chem (1962) 237:1555–62. doi: 10.1016/S0021-9258(19)83739-0 13880319

[5] Cohen S Elliott GA. The stimulation of epidermal keratinization by a protein isolated from the submaxillary gland of the mouse. J Invest Dermatol (1963) 40:1–5. doi: 10.1038/jid.1963.1 14022091

[6] Heikinheimo K Voutilainen R Happonen RP Miettinen PJ. EGF receptor and its ligands, EGF and TGF-alpha, in developing and neoplastic human odontogenic tissues. Int J Dev Biol (1993) 37:387–96. 8292533

[7] Yarden Y Sliwkowski MX. Untangling the ErbB signalling network. Nat Rev Mol Cell Biol (2001) 2:127–37. doi: 10.1038/35052073 11252954

[8] Ogiso H Ishitani R Nureki O Fukai S Yamanaka M Kim JH. Crystal structure of the complex of human epidermal growth factor and receptor extracellular domains. Cell (2002) 110:775–87. doi: 10.1016/S0092-8674(02)00963-7 12297050

[9] Ferguson KM Berger MB Mendrola JM Cho HS Leahy DJ Lemmon MA. EGF activates its receptor by removing interactions that autoinhibit ectodomain dimerization. Mol Cell (2003) 11:507–17. doi: 10.1016/S1097-2765(03)00047-9 12620237

[10] Harris RC Chung E Coffey RJ. EGF receptor ligands. Exp Cell Res (2003) 284:2–13. doi: 10.1016/S0014-4827(02)00105-2 12648462

[11] Citri A Skaria KB Yarden Y. The deaf and the dumb: the biology of ErbB-2 and ErbB-3. Exp Cell Res (2003) 284:54–65. doi: 10.1016/S0014-4827(02)00101-5 12648465

[12] Galogre M Rodin D Pyatnitskiy M Mackelprang M KOman I. A Review of HER2 overexpression and somatic mutations in cancers. Crit Rev Oncology/Hematology (2023) 103997. doi: 10.1016/j.critrevonc.2023.103997 37062337

[13] Lill NL Sever NI. Where EGF receptors transmit their signals. Sci Signal (2012) 5:pe41. doi: 10.1126/scisignal.2003341 23012653 PMC3507515

[14] Singh B Coffey RJ. Trafficking of epidermal growth factor receptor ligands in polarized epithelial cells. Annu Rev Physiol (2014) 76:275–300. doi: 10.1146/annurev-physiol-021113-170406 24215440 PMC4180094

[15] Tomas A Futter CE Eden ER. EGF receptor trafficking: consequences for signaling and cancer. Trends Cell Biol (2014) 24:26–34. doi: 10.1016/j.tcb.2013.11.002 24295852 PMC3884125

[16] Bakker J Spits M Neefjes J Berlin I. The EGFR odyssey - from activation to destruction in space and time. J Cell Sci (2017) 130:4087–96. doi: 10.1242/jcs.209197 29180516

[17] Jackman D Pao W Riely GJ Engelman JA Kris MG Janne PA. Clinical definition of acquired resistance to epidermal growth factor receptor tyrosine kinase inhibitors in non-small-cell lung cancer. J Clin Oncol (2010) 28:357–60. doi: 10.1200/JCO.2009.24.7049 PMC3870288 19949011

[18] Camidge DR Pao W Sequist LV. Acquired resistance to TKIs in solid tumours: learning from lung cancer. Nat Rev Clin Oncol (2014) 11:473–81. doi: 10.1038/nrclinonc.2014.104 24981256

[19] Santoni-Rugiu E Melchior LC Urbanska EM Jakobsen JN Stricker K Grauslund M. Intrinsic resistance to EGFR-tyrosine kinase inhibitors in EGFR-mutant non-small cell lung cancer: differences and similarities with acquired resistance. Cancers (Basel) (2019) 11. doi: 10.3390/cancers11070923 PMC6678669 31266248

[20] Lu C Mi LZ Grey MJ Zhu J Graef E Yokoyama S. Structural evidence for loose linkage between ligand binding and kinase activation in the epidermal growth factor receptor. Mol Cell Biol (2010) 30:5432–43. doi: 10.1128/MCB.00742-10 PMC2976375 20837704

[21] Mineev KS Bocharov EV Pustovalova YE Bocharova OV Chupin VV Arseniev AS. Spatial structure of the transmembrane domain heterodimer of ErbB1 and ErbB2 receptor tyrosine kinases. J Mol Biol (2010) 400:231–43. doi: 10.1016/j.jmb.2010.05.016 20471394

[22] Red Brewer M Choi SH Alvarado D Moravcevic K Pozzi A Lemmon MA. The juxtamembrane region of the EGF receptor functions as an activation domain. Mol Cell (2009) 34:641–51. doi: 10.1016/j.molcel.2009.04.034 PMC2719887 19560417

[23] Mi LZ Grey MJ Nishida N Walz T Lu C Springer TA. Functional and structural stability of the epidermal growth factor receptor in detergent micelles and phospholipid nanodiscs. Biochemistry (2008) 47:10314–23. doi: 10.1021/bi801006s PMC2658769 18771282

[24] Wang Z Longo PA Tarrant MK Kim K Head S Leahy DJ. Mechanistic insights into the activation of oncogenic forms of EGF receptor. Nat Struct Mol Biol (2011) 18:1388–93. doi: 10.1038/nsmb.2168 PMC3230693 22101934

[25] Kovacs E Zorn JA Huang Y Barros T Kuriyan J. A structural perspective on the regulation of the epidermal growth factor receptor. Annu Rev Biochem (2015) 84:739–64. doi: 10.1146/annurev-biochem-060614-034402 PMC4452390 25621509

[26] Thiel KW Carpenter G. Epidermal growth factor receptor juxtamembrane region regulates allosteric tyrosine kinase activation. Proc Natl Acad Sci U S A (2007) 104:19238–43. doi: 10.1073/pnas.0703854104 PMC2148274 18042729

[27] Jura N Endres NF Engel K Deindl S Das R Lamers MH. Mechanism for activation of the EGF receptor catalytic domain by the juxtamembrane segment. Cell (2009) 137:1293–307. doi: 10.1016/j.cell.2009.04.025 PMC2814540 19563760

[28] Chakraborty S Li L Puliyappadamba VT Guo G Hatanpaa KJ Mickey B. Constitutive and ligand-induced EGFR signalling triggers distinct and mutually exclusive downstream signalling networks. Nat Commun (2014) 5 5811. doi: 10.1038/ncomms6811 25503978 PMC4268886

[29] Byrne PO Hristova K Leahy DJ. EGFR forms ligand-independent oligomers that are distinct from the active state. J Biol Chem (2020) 295:13353–62. doi: 10.1074/jbc.RA120.012852 PMC7504936 32727847

[30] Singh B Carpenter G Coffey RJ. EGF receptor ligands: recent advances. F1000Res (2016) 5. doi: 10.12688/f1000research.9025.1 PMC5017282 27635238

[31] Orr G Hu D Ozcelik S Opresko LK Wiley HS Colson SD. Cholesterol dictates the freedom of EGF receptors and HER2 in the plane of the membrane. Biophys J (2005) 89:1362–73. doi: 10.1529/biophysj.104.056192 PMC1366621 15908575

[32] Puri C Tosoni D Comai R Rabellino A Segat D Caneva F. Relationships between EGFR signaling-competent and endocytosis-competent membrane microdomains. Mol Biol Cell (2005) 16:2704–18. doi: 10.1091/mbc.e04-07-0596 PMC1142418 15772153

[33] Hofman EG Bader AN Gerritsen HC Van Bergen En Henegouwen PM. EGF induces rapid reorganization of plasma membrane microdomains. Commun Integr Biol (2009) 2:213–4. doi: 10.4161/cib.2.3.7877 PMC2717522 19641732

[34] Irwin ME Mueller KL Bohin N Ge Y Boerner JL. Lipid raft localization of EGFR alters the response of cancer cells to the EGFR tyrosine kinase inhibitor gefitinib. J Cell Physiol (2011) 226:2316–28. doi: 10.1002/jcp.22570 PMC3103760 21660955

[35] Endres NF Das R Smith AW Arkhipov A Kovacs E Huang Y. Conformational coupling across the plasma membrane in activation of the EGF receptor. Cell (2013) 152:543–56. doi: 10.1016/j.cell.2012.12.032 PMC3718647 23374349

[36] Arkhipov A Shan Y Das R Endres NF Eastwood MP Wemmer DE. Architecture and membrane interactions of the EGF receptor. Cell (2013) 152:557–69. doi: 10.1016/j.cell.2012.12.030 PMC3680629 23374350

[37] Arkhipov A Shan Y Kim ET Shaw DE. Membrane interaction of bound ligands contributes to the negative binding cooperativity of the EGF receptor. PloS Comput Biol (2014) 10:e1003742. doi: 10.1371/journal.pcbi.1003742 25058506 PMC4109842

[38] Kumagai T Davis JG Horie T O'rourke DM Greene MI. The role of distinct p185neu extracellular subdomains for dimerization with the epidermal growth factor (EGF) receptor and EGF-mediated signaling. Proc Natl Acad Sci U S A (2001) 98:5526–31. doi: 10.1073/pnas.071060598 PMC33246 11320205

[39] Iqbal N Iqbal N. Human epidermal growth factor receptor 2 (HER2) in cancers: overexpression and therapeutic implications. Mol Biol Int (2014) 2014:852748. doi: 10.1155/2014/852748 25276427 PMC4170925

[40] Chumsri S Sperinde J Liu H Gligorov J Spano JP Antoine M. High p95HER2/HER2 ratio associated with poor outcome in trastuzumab-treated HER2-positive metastatic breast cancer NCCTG N0337 and NCCTG 98-32-52 (Alliance). Clin Cancer Res (2018) 24:3053–8. doi: 10.1158/1078-0432.CCR-17-1864 PMC6314664 29530935

[41] Carpenter G Cohen S. 125I-labeled human epidermal growth factor. Binding, internalization, and degradation in human fibroblasts. J Cell Biol (1976) 71:159–71. doi: 10.1083/jcb.71.1.159 PMC2109737 977646

[42] Sorkin A Di Fiore PP Carpenter G. The carboxyl terminus of epidermal growth factor receptor/erbB-2 chimerae is internalization impaired. Oncogene (1993) 8:3021–8. 8105439

[43] Jiang X Huang F Marusyk A Sorkin A. Grb2 regulates internalization of EGF receptors through clathrin-coated pits. Mol Biol Cell (2003) 14:858–70. doi: 10.1091/mbc.e02-08-0532 PMC151565 12631709

[44] Goh LK Huang F Kim W Gygi S Sorkin A. Multiple mechanisms collectively regulate clathrin-mediated endocytosis of the epidermal growth factor receptor. J Cell Biol (2010) 189:871–83. doi: 10.1083/jcb.201001008 PMC2878939 20513767

[45] Garay C Judge G Lucarelli S Bautista S Pandey R Singh T. Epidermal growth factor-stimulated Akt phosphorylation requires clathrin or ErbB2 but not receptor endocytosis. Mol Biol Cell (2015) 26:3504–19. doi: 10.1091/mbc.E14-09-1412 PMC4591694 26246598

[46] Sigismund S Woelk T Puri C Maspero E Tacchetti C Transidico P. Clathrin-independent endocytosis of ubiquitinated cargos. Proc Natl Acad Sci U S A (2005) 102:2760–5. doi: 10.1073/pnas.0409817102 PMC549482 15701692

[47] Pascolutti R Algisi V Conte A Raimondi A Pasham M Upadhyayula S. Molecularly distinct clathrin-coated pits differentially impact EGFR fate and signaling. Cell Rep (2019) 27:3049–61.e3046. doi: 10.1016/j.celrep.2019.05.017 31167147 PMC6581797

[48] Needham SR Roberts SK Arkhipov A Mysore VP Tynan CJ Zanetti-Domingues LC. EGFR oligomerization organizes kinase-active dimers into competent signalling platforms. Nat Commun (2016) 7:13307. doi: 10.1038/ncomms13307 27796308 PMC5095584

[49] Liang SI Van Lengerich B Eichel K Cha M Patterson DM Yoon TY. Phosphorylated EGFR dimers are not sufficient to activate Ras. Cell Rep (2018) 22:2593–600. doi: 10.1016/j.celrep.2018.02.031 PMC5916813 29514089

[50] Wiley HS Herbst JJ Walsh BJ Lauffenburger DA Rosenfeld MG Gill GN. The role of tyrosine kinase activity in endocytosis, compartmentation, and down-regulation of the epidermal growth factor receptor. J Biol Chem (1991) 266:11083–94. doi: 10.1016/S0021-9258(18)99131-3 1645724

[51] Tanaka T Zhou Y Ozawa T Okizono R Banba A Yamamura T. Ligand-activated epidermal growth factor receptor (EGFR) signaling governs endocytic trafficking of unliganded receptor monomers by non-canonical phosphorylation. J Biol Chem (2018) 293:2288–301. doi: 10.1074/jbc.M117.811299 PMC5818182 29255092

[52] Bryant DM Stow JL. Nuclear translocation of cell-surface receptors: lessons from fibroblast growth factor. Traffic (2005) 6:947–54. doi: 10.1111/j.1600-0854.2005.00332.x 16138907

[53] Wang YN Yamaguchi H Hsu JM Hung MC. Nuclear trafficking of the epidermal growth factor receptor family membrane proteins. Oncogene (2010) 29:3997–4006. doi: 10.1038/onc.2010.157 20473332 PMC2904849

[54] Vieira AV Lamaze C Schmid SL. Control of EGF receptor signaling by clathrin-mediated endocytosis. Science (1996) 274:2086–9. doi: 10.1126/science.274.5295.2086 8953040

[55] Burke P Schooler K Wiley HS. Regulation of epidermal growth factor receptor signaling by endocytosis and intracellular trafficking. Mol Biol Cell (2001) 12:1897–910. doi: 10.1091/mbc.12.6.1897 PMC37350 11408594

[56] Sorkin A Von Zastrow M. Signal transduction and endocytosis: close encounters of many kinds. Nat Rev Mol Cell Biol (2002) 3:600–14. doi: 10.1038/nrm883 12154371

[57] Fortian A Sorkin A. Live-cell fluorescence imaging reveals high stoichiometry of Grb2 binding to the EGF receptor sustained during endocytosis. J Cell Sci (2014) 127:432–44. doi: 10.1242/jcs.137786 PMC3889400 24259669

[58] Heckman CA Biswas T Dimick DM Cayer ML. Activated protein kinase C (PKC) is persistently trafficked with epidermal growth factor (EGF) receptor. Biomolecules (2020) 10. doi: 10.3390/biom10091288 PMC7563713 32906765

[59] Eden ER Huang F Sorkin A Futter CE. The role of EGF receptor ubiquitination in regulating its intracellular traffic. Traffic (2012) 13:329–37. doi: 10.1111/j.1600-0854.2011.01305.x PMC3261333 22017370

[60] Perez Verdaguer M Zhang T Surve S Paulo JA Wallace C Watkins SC. Time-resolved proximity labeling of protein networks associated with ligand-activated EGFR. Cell Rep (2022) 39:110950. doi: 10.1016/j.celrep.2022.110950 35705039 PMC9248364

[61] Sousa LP Lax I Shen H Ferguson SM De Camilli P Schlessinger J. Suppression of EGFR endocytosis by dynamin depletion reveals that EGFR signaling occurs primarily at the plasma membrane. Proc Natl Acad Sci U.S.A. (2012) 109:4419–24. doi: 10.1073/pnas.1200164109 PMC3311323 22371560

[62] Pinilla-Macua I Watkins SC Sorkin A. Endocytosis separates EGF receptors from endogenous fluorescently labeled HRas and diminishes receptor signaling to MAP kinases in endosomes. Proc Natl Acad Sci U.S.A. (2016) 113:2122–7. doi: 10.1073/pnas.1520301113 PMC4776482 26858456

[63] Surve S Watkins SC Sorkin A. EGFR-RAS-MAPK signaling is confined to the plasma membrane and associated endorecycling protrusions. J Cell Biol (2021) 220. doi: 10.1083/jcb.202107103 PMC8563293 34515735

[64] Mcnally KE Cullen PJ. Endosomal retrieval of cargo: retromer is not alone. Trends Cell Biol (2018) 28:807–22. doi: 10.1016/j.tcb.2018.06.005 30072228

[65] Steinberg F Gallon M Winfield M Thomas EC Bell AJ Heesom KJ. A global analysis of SNX27-retromer assembly and cargo specificity reveals a function in glucose and metal ion transport. Nat Cell Biol (2013) 15:461–71. doi: 10.1038/ncb2721 PMC4052425 23563491

[66] Mcnally KE Faulkner R Steinberg F Gallon M Ghai R Pim D. Retriever is a multiprotein complex for retromer-independent endosomal cargo recycling. Nat Cell Biol (2017) 19:1214–25. doi: 10.1038/ncb3610 PMC5790113 28892079

[67] Metz C Oyanadel C Jung J Retamal C Cancino J Barra J. Phosphatidic acid-PKA signaling regulates p38 and ERK1/2 functions in ligand-independent EGFR endocytosis. Traffic (2021) 22:345–61. doi: 10.1111/tra.12812 34431177

[68] Baumdick M Bruggemann Y Schmick M Xouri G Sabet O Davis L. EGF-dependent re-routing of vesicular recycling switches spontaneous phosphorylation suppression to EGFR signaling. Elife (2015) 4. doi: 10.7554/eLife.12223 PMC4716840 26609808

[69] Yammine L Zablocki A Baron W Terzi F Gallazzini M. Lipocalin-2 regulates epidermal growth factor receptor intracellular trafficking. Cell Rep (2019) 29:2067–77.e2066. doi: 10.1016/j.celrep.2019.10.015 31722218

[70] Levkowitz G Waterman H Zamir E Kam Z Oved S Langdon WY. c-Cbl/Sli-1 regulates endocytic sorting and ubiquitination of the epidermal growth factor receptor. Genes Dev (1998) 12:3663–74. doi: 10.1101/gad.12.23.3663 PMC317257 9851973

[71] Shen F Lin Q Childress C Yang W. Identification of the domain in ErbB2 that restricts ligand-induced degradation. Cell Signal (2008) 20:779–86. doi: 10.1016/j.cellsig.2007.12.021 18255265

[72] Flores-Rodriguez N Kenwright DA Chung PH Harrison AW Stefani F Waigh TA. ESCRT-0 marks an APPL1-independent transit route for EGFR between the cell surface and the EEA1-positive early endosome. J Cell Sci (2015) 128:755–67. doi: 10.1242/jcs.161786 PMC4327388 25588841

[73] Kazan JM Desrochers G Martin CE Jeong H Kharitidi D Apaja PM. Endofin is required for HD-PTP and ESCRT-0 interdependent endosomal sorting of ubiquitinated transmembrane cargoes. iScience (2021) 24:103274. doi: 10.1016/j.isci.2021.103274 34761192 PMC8567383

[74] Futter CE Pearse A Hewlett LJ Hopkins CR. Multivesicular endosomes containing internalized EGF-EGF receptor complexes mature and then fuse directly with lysosomes. J Cell Biol (1996) 132:1011–23. doi: 10.1083/jcb.132.6.1011 PMC2120766 8601581

[75] Wu L Cheng Y Geng D Fan Z Lin B Zhu Q. O-GlcNAcylation regulates epidermal growth factor receptor intracellular trafficking and signaling. Proc Natl Acad Sci U.S.A. (2022) 119:e2107453119. doi: 10.1073/pnas.2107453119 35239437 PMC8915906

[76] Cremer T Jongsma MLM Trulsson F Vertegaal ACO Neefjes J Berlin I. The ER-embedded UBE2J1/RNF26 ubiquitylation complex exerts spatiotemporal control over the endolysosomal pathway. Cell Rep (2021) 34:108659. doi: 10.1016/j.celrep.2020.108659 33472082

[77] Torrino S Tiroille V Dolfi B Dufies M Hinault C Bonesso L. UBTD1 regulates ceramide balance and endolysosomal positioning to coordinate EGFR signaling. Elife (2021) 10. doi: 10.7554/eLife.68348 PMC8118655 33884955

[78] Norris A Tammineni P Wang S Gerdes J Murr A Kwan KY. SNX-1 and RME-8 oppose the assembly of HGRS-1/ESCRT-0 degradative microdomains on endosomes. Proc Natl Acad Sci U S A (2017) 114:E307–16. doi: 10.1073/pnas.1612730114 PMC5255583 28053230

[79] Macdonald E Brown L Selvais A Liu H Waring T Newman D. HRS-WASH axis governs actin-mediated endosomal recycling and cell invasion. J Cell Biol (2018) 217:2549–64. doi: 10.1083/jcb.201710051 PMC6028553 29891722

[80] Raiborg C Wesche J Malerod L Stenmark H. Flat clathrin coats on endosomes mediate degradative protein sorting by scaffolding Hrs in dynamic microdomains. J Cell Sci (2006) 119:2414–24. doi: 10.1242/jcs.02978 16720641

[81] Wenzel EM Schultz SW Schink KO Pedersen NM Nahse V Carlson A. Concerted ESCRT and clathrin recruitment waves define the timing and morphology of intraluminal vesicle formation. Nat Commun (2018) 9:2932. doi: 10.1038/s41467-018-05345-8 30050131 PMC6062606

[82] Hu S Li B Wu F Zhu D Zouhar J Gao C. Plant ESCRT protein ALIX coordinates with retromer complex in regulating receptor-mediated sorting of soluble vacuolar proteins. Proc Natl Acad Sci U S A (2022) 119:e2200492119. doi: 10.1073/pnas.2200492119 35533279 PMC9171914

[83] Iqbal N Iqbal N. Imatinib: a breakthrough of targeted therapy in cancer. Chemother Res Pract (2014) 2014:357027. doi: 10.1155/2014/357027 24963404 PMC4055302

[84] He J Huang Z Han L Gong Y Xie C. Mechanisms and management of 3rd−generation EGFR−TKI resistance in advanced non−small cell lung cancer (Review). Int J Oncol (2021) 59. doi: 10.3892/ijo.2021.5270 PMC8562388 34558640

[85] Kazandjian D Blumenthal GM Yuan W He K Keegan P Pazdur R. FDA approval of gefitinib for the treatment of patients with metastatic EGFR mutation-positive non-small cell lung cancer. Clin Cancer Res (2016) 22:1307–12. doi: 10.1158/1078-0432.CCR-15-2266 26980062

[86] Rosell R Carcereny E Gervais R Vergnenegre A Massuti B Felip E. Erlotinib versus standard chemotherapy as first-line treatment for European patients with advanced EGFR mutation-positive non-small-cell lung cancer (EURTAC): a multicentre, open-label, randomised phase 3 trial. Lancet Oncol (2012) 13:239–46. doi: 10.1016/S1470-2045(11)70393-X 22285168

[87] Opdam FL Guchelaar HJ Beijnen JH Schellens JH. Lapatinib for advanced or metastatic breast cancer. Oncologist (2012) 17:536–42. doi: 10.1634/theoncologist.2011-0461 PMC3336826 22477724

[88] Park K Tan EH O'byrne K Zhang L Boyer M Mok T. Afatinib versus gefitinib as first-line treatment of patients with EGFR mutation-positive non-small-cell lung cancer (LUX-Lung 7): a phase 2B, open-label, randomised controlled trial. Lancet Oncol (2016) 17:577–89. doi: 10.1016/S1470-2045(16)30033-X 27083334

[89] Wu YL Cheng Y Zhou X Lee KH Nakagawa K Niho S. Dacomitinib versus gefitinib as first-line treatment for patients with EGFR-mutation-positive non-small-cell lung cancer (ARCHER 1050): a randomised, open-label, phase 3 trial. Lancet Oncol (2017) 18:1454–66. doi: 10.1016/S1470-2045(17)30608-3 28958502

[90] Westover D Zugazagoitia J Cho BC Lovly CM Paz-Ares L. Mechanisms of acquired resistance to first- and second-generation EGFR tyrosine kinase inhibitors. Ann Oncol (2018) 29:i10–9. doi: 10.1093/annonc/mdx703 PMC6454547 29462254

[91] Deeks ED. Neratinib: first global approval. Drugs (2017) 77:1695–704. doi: 10.1007/s40265-017-0811-4 28884417

[92] Jackisch C Barcenas CH Bartsch R Palma JD Gluck S Harbeck N. Optimal strategies for successful initiation of neratinib in patients with HER2-positive breast cancer. Clin Breast Cancer (2021) 21:e575–83. doi: 10.1016/j.clbc.2021.02.001 33678567

[93] Eli LD Kavuri SM. Mechanisms of neratinib resistance in HER2-mutant metastatic breast cancer. Cancer Drug Resist (2022) 5:873–81. doi: 10.20517/cdr.2022.48 PMC9771739 36627899

[94] Li R Zhou X Yao H Li L. Four generations of EGFR TKIs associated with different pathogenic mutations in non-small cell lung carcinoma. J Drug Target (2020) 28:861–72. doi: 10.1080/1061186X.2020.1737934 32118494

[95] Sequist LV Soria JC Camidge DR. Update to rociletinib data with the RECIST confirmed response rate. N Engl J Med (2016) 374:2296–7. doi: 10.1056/NEJMc1602688 27195670

[96] RaMalingam SS Vansteenkiste J Planchard D Cho BC Gray JE Ohe Y. Overall survival with osimertinib in untreated, EGFR-mutated advanced NSCLC. N Engl J Med (2020) 382:41–50. doi: 10.1056/NEJMoa1913662 31751012

[97] Reungwetwattana T Nakagawa K Cho BC Cobo M Cho EK Bertolini A. CNS response to osimertinib versus standard epidermal growth factor receptor tyrosine kinase inhibitors in patients with untreated EGFR-mutated advanced non-small-cell lung cancer. J Clin Oncol (2018) 36:3290–3297. doi: 10.1200/JCO.2018.78.3118 30153097

[98] Passaro A Guerini-Rocco E Pochesci A Vacirca D Spitaleri G Catania CM. Targeting EGFR T790M mutation in NSCLC: From biology to evaluation and treatment. Pharmacol Res (2017) 117:406–15. doi: 10.1016/j.phrs.2017.01.003 28089942

[99] Duggirala KB Lee Y Lee K. Chronicles of EGFR tyrosine kinase inhibitors: targeting EGFR C797S containing triple mutations. Biomol Ther (Seoul) (2022) 30:19–27. doi: 10.4062/biomolther.2021.047 34074804 PMC8724843

[100] Xiao Z Huang X Xie B Xie W Huang M Lin L. Primary resistance to brigatinib in a patient with lung adenocarcinoma harboring ALK G1202R mutation and LIPI-NTRK1 rearrangement. Onco Targets Ther (2020) 13:4591–5. doi: 10.2147/OTT.S249652 PMC7250292 32547089

[101] Commander H Whiteside G Perry C. Vandetanib: first global approval. Drugs (2011) 71:1355–65. doi: 10.2165/11595310-000000000-00000 21770481

[102] Chau NG Haddad RI. Vandetanib for the treatment of medullary thyroid cancer. Clin Cancer Res (2013) 19:524–9. doi: 10.1158/1078-0432.CCR-12-2353 23231950

[103] Nakaoku T Kohno T Araki M Niho S Chauhan R Knowles PP. A secondary RET mutation in the activation loop conferring resistance to vandetanib. Nat Commun (2018) 9:625. doi: 10.1038/s41467-018-02994-7 29434222 PMC5809600

[104] Roskoski R Jr. Properties of FDA-approved small molecule protein kinase inhibitors. Pharmacol Res (2019) 144:19–50. doi: 10.1016/j.phrs.2019.03.006 30877063

[105] Pottier C Fresnais M Gilon M Jerusalem G Longuespee R Sounni NE. Tyrosine kinase inhibitors in cancer: breakthrough and challenges of targeted therapy. Cancers (Basel) (2020) 12. doi: 10.3390/cancers12030731 PMC7140093 32244867

[106] Yu HA Arcila ME Rekhtman N Sima CS Zakowski MF Pao W. Analysis of tumor specimens at the time of acquired resistance to EGFR-TKI therapy in 155 patients with EGFR-mutant lung cancers. Clin Cancer Res (2013) 19:2240–7. doi: 10.1158/1078-0432.CCR-12-2246 PMC3630270 23470965

[107] Eberwein P Laird D Schulz S Reinhard T Steinberg T Tomakidi P. Modulation of focal adhesion constituents and their down-stream events by EGF: On the cross-talk of integrins and growth factor receptors. Biochim Biophys Acta (2015) 1853:2183–98. doi: 10.1016/j.bbamcr.2015.06.004 26079101

[108] Huafeng J Deqing Z Yong D Yulian Z Ailing H. A cross-talk between integrin beta4 and epidermal growth factor receptor induces gefitinib chemoresistance to gastric cancer. Cancer Cell Int (2018) 18:50. doi: 10.1186/s12935-018-0548-5 29618949 PMC5879569

[109] Cross DA Ashton SE Ghiorghiu S Eberlein C Nebhan CA Spitzler PJ. AZD9291, an irreversible EGFR TKI overcomes T790M-mediated resistance to EGFR inhibitors in lung cancer. Cancer Discovery (2014) 4:1046–61. doi: 10.1158/2159-8290.CD-14-0337 PMC4315625 24893891

[110] Thress KS Paweletz CP Felip E Cho BC Stetson D Dougherty B. Acquired EGFR C797S mutation mediates resistance to AZD9291 in non-small cell lung cancer harboring EGFR T790M. Nat Med (2015) 21:560–2. doi: 10.1038/nm.3854 PMC4771182 25939061

[111] Rodriguez SMB Kamel A Ciubotaru GV Onose G Sevastre A-S Sfredel V. An overview of EGFR mechanisms and their implications in targeted therapies for glioblastoma. Int J Mol Sci (2023) 24:11110. doi: 10.3390/ijms241311110 37446288 PMC10341823

[112] Schlam I Swain SM. HER2-positive breast cancer and tyrosine kinase inhibitors: the time is now. NPJ Breast Cancer (2021) 7:56. doi: 10.1038/s41523-021-00265-1 34016991 PMC8137941

[113] Strickler JH Cercek A Siena S Andre T Ng K Van Cutsem E. Tucatinib plus trastuzumab for chemotherapy-refractory, HER2-positive, RAS wild-type unresectable or metastatic colorectal cancer (MOUNTAINEER): a multicentre, open-label, phase 2 study. Lancet Oncol (2023) 24:496–508. doi: 10.1016/S1470-2045(23)00150-X 37142372

[114] Chen D Song Z Cheng G. Clinical efficacy of first-generation EGFR-TKIs in patients with advanced non-small-cell lung cancer harboring EGFR exon 20 mutations. Onco Targets Ther (2016) 9:4181–6. doi: 10.2147/OTT.S108242 PMC4944908 27468240

[115] Rao DS Bradley SV Kumar PD Hyun TS Saint-Dic D Oravecz-Wilson K. Altered receptor trafficking in Huntingtin Interacting Protein 1-transformed cells. Cancer Cell (2003) 3:471–82. doi: 10.1016/S1535-6108(03)00107-7 12781365

[116] Chung BM Raja SM Clubb RJ Tu C George M Band V. Aberrant trafficking of NSCLC-associated EGFR mutants through the endocytic recycling pathway promotes interaction with Src. BMC Cell Biol (2009) 10:84. doi: 10.1186/1471-2121-10-84 19948031 PMC2790444

[117] Cao X Zhu H Ali-Osman F Lo HW. EGFR and EGFRvIII undergo stress- and EGFR kinase inhibitor-induced mitochondrial translocalization: a potential mechanism of EGFR-driven antagonism of apoptosis. Mol Cancer (2011) 10:26. doi: 10.1186/1476-4598-10-26 21388543 PMC3063231

[118] Boerner JL Danielsen A Maihle NJ. Ligand-independent oncogenic signaling by the epidermal growth factor receptor: v-ErbB as a paradigm. Exp Cell Res (2003) 284:111–21. doi: 10.1016/S0014-4827(02)00096-4 12648470

[119] Donepudi M Resh MD. c-Src trafficking and co-localization with the EGF receptor promotes EGF ligand-independent EGF receptor activation and signaling. Cell Signal (2008) 20:1359–67. doi: 10.1016/j.cellsig.2008.03.007 PMC2459337 18448311

[120] Roepstorff K Grovdal L Grandal M Lerdrup M Van Deurs B. Endocytic downregulation of ErbB receptors: mechanisms and relevance in cancer. Histochem Cell Biol (2008) 129:563–78. doi: 10.1007/s00418-008-0401-3 PMC2323030 18288481

[121] Shan Y Eastwood MP Zhang X Kim ET Arkhipov A Dror RO. Oncogenic mutations counteract intrinsic disorder in the EGFR kinase and promote receptor dimerization. Cell (2012) 149:860–70. doi: 10.1016/j.cell.2012.02.063 22579287

[122] Davies GC Ryan PE Rahman L Zajac-Kaye M Lipkowitz S. EGFRvIII undergoes activation-dependent downregulation mediated by the Cbl proteins. Oncogene (2006) 25:6497–509. doi: 10.1038/sj.onc.1209662 PMC2274962 16702950

[123] Gan HK Kaye AH Luwor RB. The EGFRvIII variant in glioblastoma multiforme. J Clin Neurosci (2009) 16:748–54. doi: 10.1016/j.jocn.2008.12.005 19324552

[124] Huang F Kirkpatrick D Jiang X Gygi S Sorkin A. Differential regulation of EGF receptor internalization and degradation by multiubiquitination within the kinase domain. Mol Cell (2006) 21:737–48. doi: 10.1016/j.molcel.2006.02.018 16543144

[125] Li A Zhang C Gao S Chen F Yang C Luo R. TIP30 loss enhances cytoplasmic and nuclear EGFR signaling and promotes lung adenocarcinogenesis in mice. Oncogene (2013) 32:2273–2281, 2281e 2271-2212. doi: 10.1038/onc.2012.253 22733137 PMC3460142

[126] Shuai S Liao X Wang H Liu L Mei S Cao J. TIP30 overcomes gefitinib resistance by regulating cytoplasmic and nuclear EGFR signaling in non-small-cell lung cancer. Cancer Sci (2021) 112:4139–50. doi: 10.1111/cas.15000 PMC8486181 34058054

[127] Hsu SC Hung MC. Characterization of a novel tripartite nuclear localization sequence in the EGFR family. J Biol Chem (2007) 282:10432–40. doi: 10.1074/jbc.M610014200 17283074

[128] Agus DB Akita RW Fox WD Lewis GD Higgins B Pisacane PI. Targeting ligand-activated ErbB2 signaling inhibits breast and prostate tumor growth. Cancer Cell (2002) 2:127–37. doi: 10.1016/S1535-6108(02)00097-1 12204533

[129] Arteaga CL. Can trastuzumab be effective against tumors with low HER2/Neu (ErbB2) receptors? J Clin Oncol (2006) 24:3722–5. doi: 10.1200/JCO.2006.06.5268 16847283

[130] Lee-Hoeflich ST Crocker L Yao E Pham T Munroe X Hoeflich KP. A central role for HER3 in HER2-amplified breast cancer: implications for targeted therapy. Cancer Res (2008) 68:5878–87. doi: 10.1158/0008-5472.CAN-08-0380 18632642

[131] Hendriks BS Opresko LK Wiley HS Lauffenburger D. Coregulation of epidermal growth factor receptor/human epidermal growth factor receptor 2 (HER2) levels and locations: quantitative analysis of HER2 overexpression effects. Cancer Res (2003) 63:1130–7. 12615732

[132] Austin CD De Maziere AM Pisacane PI Van Dijk SM Eigenbrot C Sliwkowski MX. Endocytosis and sorting of ErbB2 and the site of action of cancer therapeutics trastuzumab and geldanamycin. Mol Biol Cell (2004) 15:5268–82. doi: 10.1091/mbc.e04-07-0591 PMC532009 15385631

[133] Citri A Gan J Mosesson Y Vereb G Szollosi J Yarden Y. Hsp90 restrains ErbB-2/HER2 signalling by limiting heterodimer formation. EMBO Rep (2004) 5:1165–70. doi: 10.1038/sj.embor.7400300 PMC1299195 15568014

[134] Longva KE Pedersen NM Haslekas C Stang E Madshus IH. Herceptin-induced inhibition of ErbB2 signaling involves reduced phosphorylation of Akt but not endocytic down-regulation of ErbB2. Int J Cancer (2005) 116:359–67. doi: 10.1002/ijc.21015 15800944

[135] Bertelsen V Stang E. The mysterious ways of ErbB2/HER2 trafficking. Membranes (Basel) (2014) 4:424–46. doi: 10.3390/membranes4030424 PMC4194043 25102001

[136] Worthylake R Opresko LK Wiley HS. ErbB-2 amplification inhibits down-regulation and induces constitutive activation of both ErbB-2 and epidermal growth factor receptors. J Biol Chem (1999) 274:8865–74. doi: 10.1074/jbc.274.13.8865 10085130

[137] Raina D Uchida Y Kharbanda A Rajabi H Panchamoorthy G Jin C. Targeting the MUC1-C oncoprotein downregulates HER2 activation and abrogates trastuzumab resistance in breast cancer cells. Oncogene (2014) 33:3422–31. doi: 10.1038/onc.2013.308 PMC3916940 23912457

[138] Hommelgaard AM Lerdrup M Van Deurs B. Association with membrane protrusions makes ErbB2 an internalization-resistant receptor. Mol Biol Cell (2004) 15:1557–67. doi: 10.1091/mbc.e03-08-0596 PMC379255 14742716

[139] Cortese K Howes MT Lundmark R Tagliatti E Bagnato P Petrelli A. The HSP90 inhibitor geldanamycin perturbs endosomal structure and drives recycling ErbB2 and transferrin to modified MVBs/lysosomal compartments. Mol Biol Cell (2013) 24:129–44. doi: 10.1091/mbc.e12-04-0282 PMC3541960 23154999

[140] Haslekas C Breen K Pedersen KW Johannessen LE Stang E Madshus IH. The inhibitory effect of ErbB2 on epidermal growth factor-induced formation of clathrin-coated pits correlates with retention of epidermal growth factor receptor-ErbB2 oligomeric complexes at the plasma membrane. Mol Biol Cell (2005) 16:5832–42. doi: 10.1091/mbc.e05-05-0456 PMC1289425 16207817

[141] Lerdrup M Bruun S Grandal MV Roepstorff K Kristensen MM Hommelgaard AM. Endocytic down-regulation of ErbB2 is stimulated by cleavage of its C-terminus. Mol Biol Cell (2007) 18:3656–66. doi: 10.1091/mbc.e07-01-0025 PMC1951740 17626164

[142] Pedersen NM Madshus IH Haslekas C Stang E. Geldanamycin-induced down-regulation of ErbB2 from the plasma membrane is clathrin dependent but proteasomal activity independent. Mol Cancer Res (2008) 6:491–500. doi: 10.1158/1541-7786.MCR-07-0191 18337455

[143] Asp N Pust S Sandvig K. Flotillin depletion affects ErbB protein levels in different human breast cancer cells. Biochim Biophys Acta (2014) 1843:1987–96. doi: 10.1016/j.bbamcr.2014.04.013 24747692

[144] Li Q Ma W Li T. Sortilin as a new membrane inhibitor of EGFR trafficking for overcoming resistance to EGFR inhibitors in non-small cell lung cancer. J Thorac Dis (2018) 10:S3186–91. doi: 10.21037/jtd.2018.08.25 PMC6186589 30430029

[145] Pietila M Sahgal P Peuhu E Jantti NZ Paatero I Narva E. SORLA regulates endosomal trafficking and oncogenic fitness of HER2. Nat Commun (2019) 10:2340. doi: 10.1038/s41467-019-10275-0 31138794 PMC6538630

[146] Xu W Yuan X Xiang Z Mimnaugh E Marcu M Neckers L. Surface charge and hydrophobicity determine ErbB2 binding to the Hsp90 chaperone complex. Nat Struct Mol Biol (2005) 12:120–6. doi: 10.1038/nsmb885 15643424

[147] Sidera K Gaitanou M Stellas D Matsas R Patsavoudi E. A critical role for HSP90 in cancer cell invasion involves interaction with the extracellular domain of HER-2. J Biol Chem (2008) 283:2031–41. doi: 10.1074/jbc.M701803200 18056992

[148] Kancha RK Bartosch N Duyster J. Analysis of conformational determinants underlying HSP90-kinase interaction. PloS One (2013) 8:e68394. doi: 10.1371/journal.pone.0068394 23844194 PMC3699556

[149] Mishra A Hourigan D Lindsay AJ. Inhibition of the endosomal recycling pathway downregulates HER2 activation and overcomes resistance to tyrosine kinase inhibitors in HER2-positive breast cancer. Cancer Lett (2022) 529:153–67. doi: 10.1016/j.canlet.2022.01.003 35007696

[150] Molina MA Codony-Servat J Albanell J Rojo F Arribas J Baselga J. Trastuzumab (herceptin), a humanized anti-Her2 receptor monoclonal antibody, inhibits basal and activated Her2 ectodomain cleavage in breast cancer cells. Cancer Res (2001) 61:4744–9. 11406546

[151] Junttila TT Akita RW Parsons K Fields C Lewis Phillips GD Friedman LS. Ligand-independent HER2/HER3/PI3K complex is disrupted by trastuzumab and is effectively inhibited by the PI3K inhibitor GDC-0941. Cancer Cell (2009) 15:429–40. doi: 10.1016/j.ccr.2009.03.020 19411071

[152] Ram S Kim D Ober RJ Ward ES. The level of HER2 expression is a predictor of antibody-HER2 trafficking behavior in cancer cells. MAbs (2014) 6:1211–9. doi: 10.4161/mabs.29865 PMC4622696 25517306

[153] Fehling-Kaschek M Peckys DB Kaschek D Timmer J Jonge N. Mathematical modeling of drug-induced receptor internalization in the HER2-positive SKBR3 breast cancer cell-line. Sci Rep (2019) 9:12709. doi: 10.1038/s41598-019-49019-x 31481718 PMC6722142

[154] Scaltriti M Rojo F Ocana A Anido J Guzman M Cortes J. Expression of p95HER2, a truncated form of the HER2 receptor, and response to anti-HER2 therapies in breast cancer. J Natl Cancer Inst (2007) 99:628–38. doi: 10.1093/jnci/djk134 17440164

[155] Bao W Fu HJ Jia LT Zhang Y Li W Jin BQ. HER2-mediated upregulation of MMP-1 is involved in gastric cancer cell invasion. Arch Biochem Biophys (2010) 499:49–55. doi: 10.1016/j.abb.2010.05.009 20460098

[156] Zagozdzon R Gallagher WM Crown J. Truncated HER2: implications for HER2-targeted therapeutics. Drug Discovery Today (2011) 16:810–6. doi: 10.1016/j.drudis.2011.06.003 21704182

[157] Tse C Gauchez AS Jacot W Lamy PJ. HER2 shedding and serum HER2 extracellular domain: biology and clinical utility in breast cancer. Cancer Treat Rev (2012) 38:133–42. doi: 10.1016/j.ctrv.2011.03.008 21549508

[158] Sperinde J Jin X Banerjee J Penuel E Saha A Diedrich G. Quantitation of p95HER2 in paraffin sections by using a p95-specific antibody and correlation with outcome in a cohort of trastuzumab-treated breast cancer patients. Clin Cancer Res (2010) 16:4226–35. doi: 10.1158/1078-0432.CCR-10-0410 20664024

[159] Cappuzzo F Cho YG Sacconi A Ali G Siclari O Incarbone M. p95HER2 truncated form in resected non-small cell lung cancer. J Thorac Oncol (2012) 7:520–7. doi: 10.1097/JTO.0b013e318249e13f PMC4608748 22307009

[160] Han SW Cha Y Paquet A Huang W Weidler J Lie Y. Correlation of HER2, p95HER2 and HER3 expression and treatment outcome of lapatinib plus capecitabine in her2-positive metastatic breast cancer. PloS One (2012) 7:e39943. doi: 10.1371/journal.pone.0039943 22848366 PMC3407213

[161] Franklin MC Carey KD Vajdos FF Leahy DJ De Vos AM Sliwkowski MX. Insights into ErbB signaling from the structure of the ErbB2-pertuzumab complex. Cancer Cell (2004) 5:317–28. doi: 10.1016/S1535-6108(04)00083-2 15093539

[162] Rufo M. ["Do you want a little brother or a little sister?"]. Ann Pediatr (Paris) (1988) 35:694–5. 3195976

[163] Friedman LM Rinon A Schechter B Lyass L Lavi S Bacus SS. Synergistic down-regulation of receptor tyrosine kinases by combinations of mAbs: implications for cancer immunotherapy. Proc Natl Acad Sci U S A (2005) 102:1915–20. doi: 10.1073/pnas.0409610102 PMC548578 15684082

[164] Zhu W Okollie B Artemov D. Controlled internalization of Her-2/ neu receptors by cross-linking for targeted delivery. Cancer Biol Ther (2007) 6:1960–1966.0. doi: 10.4161/cbt.6.12.4979 18075296

[165] Ren XR Wei J Lei G Wang J Lu J Xia W. Polyclonal HER2-specific antibodies induced by vaccination mediate receptor internalization and degradation in tumor cells. Breast Cancer Res (2012) 14:R89. doi: 10.1186/bcr3204 22676470 PMC3446352

[166] Leyton JV. Improving receptor-mediated intracellular access and accumulation of antibody therapeutics-the tale of HER2. Antibodies (Basel) (2020) 9. doi: 10.3390/antib9030032 PMC7551051 32668710

[167] Szymanska M Fosdahl AM Nikolaysen F Pedersen MW Grandal MM Stang E. A combination of two antibodies recognizing non-overlapping epitopes of HER2 induces kinase activity-dependent internalization of HER2. J Cell Mol Med (2016) 20:1999–2011. doi: 10.1111/jcmm.12899 27469139 PMC5020627

[168] Cheng J Liang M Carvalho MF Tigue N Faggioni R Roskos LK. Molecular mechanism of HER2 rapid internalization and redirected trafficking induced by anti-HER2 biparatopic antibody. Antibodies (Basel) (2020) 9. doi: 10.3390/antib9030049 PMC7551206 32961882

[169] Tiwari SR Mishra P Abraham J. Neratinib, A novel HER2-targeted tyrosine kinase inhibitor. Clin Breast Cancer (2016) 16:344–8. doi: 10.1016/j.clbc.2016.05.016 27405796

[170] Santamaria S Gagliani MC Bellese G Marconi S Lechiara A Dameri M. Imaging of endocytic trafficking and extracellular vesicles released under neratinib treatment in ERBB2(+) breast cancer cells. J Histochem Cytochem (2021) 69:461–73. doi: 10.1369/00221554211026297 PMC8246527 34126793

[171] Jo U Park KH Whang YM Sung JS Won NH Park JK. EGFR endocytosis is a novel therapeutic target in lung cancer with wild-type EGFR. Oncotarget (2014) 5:1265–78. doi: 10.18632/oncotarget.1711 PMC4012721 24658031

[172] Kim B Park YS Sung JS Lee JW Lee SB Kim YH. Clathrin-mediated EGFR endocytosis as a potential therapeutic strategy for overcoming primary resistance of EGFR TKI in wild-type EGFR non-small cell lung cancer. Cancer Med (2021) 10:372–85. doi: 10.1002/cam4.3635 PMC7826488 33314735

[173] Menard L Floc'h N Martin MJ Cross D. Reactivation of mutant-EGFR degradation through clathrin inhibition overcomes resistance to EGFR tyrosine kinase inhibitors. Cancer Res (2018) 78:3267–79. doi: 10.1158/0008-5472.CAN-17-2195 29555874

[174] Yao N Wang CR Liu MQ Li YJ Chen WM Li ZQ. Discovery of a novel EGFR ligand DPBA that degrades EGFR and suppresses EGFR-positive NSCLC growth. Signal Transduct Target Ther (2020) 5:214. doi: 10.1038/s41392-020-00251-2 33033232 PMC7544691

[175] Rush JS Quinalty LM Engelman L Sherry DM Ceresa BP. Endosomal accumulation of the activated epidermal growth factor receptor (EGFR) induces apoptosis. J Biol Chem (2012) 287:712–22. doi: 10.1074/jbc.M111.294470 PMC3249126 22102283

[176] Dutta S Roy S Polavaram NS Stanton MJ Zhang H Bhola T. Neuropilin-2 regulates endosome maturation and EGFR trafficking to support cancer cell pathobiology. Cancer Res (2016) 76:418–28. doi: 10.1158/0008-5472.CAN-15-1488 PMC4715955 26560516

[177] Cordero JB Cozzolino M Lu Y Vidal M Slatopolsky E Stahl PD. 1,25-Dihydroxyvitamin D down-regulates cell membrane growth- and nuclear growth-promoting signals by the epidermal growth factor receptor. J Biol Chem (2002) 277:38965–71. doi: 10.1074/jbc.M203736200 12181310

[178] Kim JH Choi HS Lee DS. Primaquine inhibits the endosomal trafficking and nuclear localization of EGFR and induces the apoptosis of breast cancer cells by nuclear EGFR/Stat3-mediated c-Myc downregulation. Int J Mol Sci (2021) 22. doi: 10.3390/ijms222312961 PMC8657416 34884765

